# Cartilage Protection and Analgesic Activity of a Botanical Composition Comprised of* Morus alba*,* Scutellaria baicalensis*, and* Acacia catechu*

**DOI:** 10.1155/2017/7059068

**Published:** 2017-08-20

**Authors:** Mesfin Yimam, Teresa Horm, Laura Wright, Ping Jiao, Mei Hong, Lidia Brownell, Qi Jia

**Affiliations:** Unigen Inc., 3005 1st Ave., Seattle, WA 98121, USA

## Abstract

Although there have been augmented advances in drug discovery, current OA management is inadequate due to the lack of successful therapies proven to be effective in modifying disease progression. For some, the risk outweighs the benefit. As a result, there is a desperate need for safe and efficacious natural alternatives. Here we evaluated a composition from* Morus alba*,* Scutellaria baicalensis*, and* Acacia catechu* in maintaining joint structural integrity and alleviating OA associated symptoms in monoiodoacetate- (MIA-) induced rat OA disease model. Study lasted for 6 weeks. 59.6%, 64.6%, 70.7%, 69.9%, and 70.3% reductions in pain sensitivity were observed for rats treated with the composition from week 1 to week 5, respectively. Statistically significant improvements in articular cartilage matrix integrity (maintained at 57.1% versus MIA + vehicle treated rats) were shown from the modified total Mankin score for animals treated with the composition. The composition showed a statistically significant reduction in uCTX-II level (54.1% reductions). The merit of combining these botanicals was also demonstrated in their synergistic analgesic activity. Therefore, the standardized blend of* Morus alba*,* Scutellaria baicalensis*, and* Acacia catechu* could potentially be considered as an alternative remedy from natural sources for the management of OA and/or its associated symptoms.

## 1. Introduction

Osteoarthritis (OA), the most common form of arthritis, is characterized by progressive articular cartilage degradation associated with joint pain and dysfunction impacting an estimated 30.8-million adult population in the US [1]. In spite of the fact that there have been augmented advances in drug discovery, current pharmaceutical based OA management is inadequate due to the lack of successful therapies proven to be effective in modifying disease progression. The present day approach which mainly focuses on symptomatic relief (using over-the-counter nonsteroidal anti-inflammatory drugs, NSAIDs) will most likely mask the primary etiology leading to irreversible damage to the articular structure. In addition, chronic uses of NSAIDs for symptomatic relief of OA are also limited due to their gastrointestinal, renal, and cardiovascular side effects [2]. As a result, there is a desperate need for a safe and efficacious natural alternative from medicinal plants.

It is believed that at various stages of OA all the three major structures of the joint (cartilage, subchondral bone, and synovium) could be involved in the pathophysiology of the disease which complicates the identification of a single biomarker that is crucial for immediate therapeutic intervention at the early stage of the disease. Nevertheless, among all the major joint biomarkers proposed, C-terminal telopeptide of type II collagen (CTX-II) has been by far the most studied and frequently referred biomarker of cartilage degradation that could be used for the purpose of diagnosis, determining severity of disease or extent of disease progression, prognosis, and monitoring efficacy of treatment. It is primarily generated by matrix metalloproteinase activity during cartilage degradation in OA. It is known to show a closer link with progression of articular cartilage degradation in OA patients [3, 4]. Its increased serum, urine or synovial fluid levels, and correlations with articular cartilage degradation were reported in both preclinical and clinical studies [3, 4] suggesting plant extracts with inherent characteristic of reducing CTX-II level to be also potentially utilized for their cartilage protection activity.

Recently we reported a botanical composition designated as UP1306 (a proprietary blend of two bioflavonoid standardized extracts from the heartwood of* Acacia catechu* and root bark of* Morus alba*) with analgesic and anti-inflammatory effects [5]. UP1306 resulted in 46.3%–53.3% reductions in paw edema and 43.6%–54.8% reductions in pain sensitivity in the carrageenan induced rat paw edema model as well as a 34.4% reduction in visceral pain sensitivity in Writhing's model in mice [5]. IC_50_ (concentration causing 50% inhibition) values of 20.9 *μ*g/mL, 49.2 *μ*g/mL, and 11.1 *μ*g/mL in cyclooxygenase 1 (COX-1), cyclooxygenase 2 (COX-2), and 5-lipoxygenase (5-LOX), respectively, were documented for UP1306 [5]. Collectively, these findings suggest that UP1306 may be considered for OA management.

Previously we have also documented that UP446 consists primarily of baicalin from* Scutellaria baicalensis* and catechin from* Acacia catechu* to possess activities suggestive of benefit in OA including (i) dual inhibition of COX and LOX (Burnett et al., 2007), (ii) normalization of COX-2, tumor necrosis factor-*α* (TNF-*α*), IL-1*β*, IL-6, and nuclear factor-*κ*B (NF-*κ*B) gene expression in lipopolysaccharide- (LPS-) induced human and animal cell lines (Tseng-Crank et al., 2010), and (iii) inhibition of COX-2, 5-LOX, and inducible-nitric oxide synthase (iNOS) gene expression and moderation of NF-*κ*B binding activity in endotoxin-stimulated rat peritoneal macrophages (Altavilla et al., 2009). Beneficial applications in OA related symptomatic pain relief had also been reported for UP446 from human clinical trials [6, 7]. However, its usage in support for cartilage protection has not yet been reported.

Given the above facts, we postulated that the coadministration of these materials at a specific ratio could provide cartilage protection activity while reducing associated symptoms and we proceeded to test the hypothesis in an animal model that mimics the human type of osteoarthritis. Hence, present study was designed to evaluate the potential analgesic and/or cartilage degradation protection activity of UP1306 and UP446 alone or in a standardized blend using a monoiodoacetate- (MIA-) induced OA disease model in rats.

## 2. Materials and Methods

### 2.1. Preparation of the Composition UP1306

Dried* Morus alba* root barks were cut, crushed, and then extracted with approximately sevenfold volume of 70% ethyl alcohol in water at 100°C for 4 hrs three times to give* Morus alba* 70% EtOH extract powder with a yield of 19.6% (w/w). The standardized* Morus* extract contains no less than 4% mulberroside A and no less than 3% of total bioflavonoids including kuwanon G, albanin G, and morusin. Catechins enriched* Acacia* extract was obtained by repeated crystallization from the aqueous extract of* Acacia catechu* heartwood with a yield of 14.5% (w/w). (+)-Catechin was identified as the major active flavan in the* A. catechu* extract. The* Acacia* extract was standardized as no less than 65% of catechin and a minor enantiomer epicatechin. Composition UP1306 was prepared by mixing the standardized extracts of* Acacia catechu* heartwood (no less than 65% catechins) and* Morus alba* root bark (no less than 7% stilbenes and bioflavonoids) at a ratio of 1 : 2 by weight with no less than 15% catechins and 2% stilbenes and bioflavonoids.

### 2.2. Preparation of the Composition UP446

The* Scutellaria baicalensis* extract was prepared by extraction of dried* Scutellaria baicalensis* roots powder with water and then recrystallization to give the final product containing baicalin as the major bioflavonoid at content not less than 75% as well as other minor free-B-ring flavonoids such as wogonin-7-O–glucuronide, oroxylin A-7-O- glucuronide, and baicalein.* Acacia* extract was obtained from repeated crystallization of an aqueous extract of the heartwoods of an India medicinal plant,* A. catechu*. (+)-Catechin is the major component in the* A. catechu* extract with a content of not less than 65% plus a minor amount of its enantiomer and epicatechin, as well as other minor amounts of flavans. Composition UP446 was a mixture of the* S. baicalensis* and* A. catechu* standardized extracts at a ratio 4 : 1 with baicalin content not less than 60% and catechin content not less than 10%. Other minor flavonoids, such as wogonin 7-glucuronide and baicalein, account for about 15% of total weight.

Through the years, we have documented significant efficacy and toxicity data for UP446 (with* Scutellaria* and* Acacia* at 4 : 1 ratio) and UP1306 (with* Acacia* and* Morus* at 1 : 2 ratio) at oral doses of 250 mg/kg and 400 mg/kg, respectively. Their standardized blend was tested for efficacy at 650 mg/kg (250 + 400) yielding a composition with* Acacia* (A),* Scutellaria* (S), and* Morus* (M) extracts at the ratio of 0.282A : 0.308S : 0.410M by weight.

### 2.3. Animals

Sprague-Dawley rats were purchased form Charles River Laboratories (Hollister, CA, USA) at the age of 8 weeks and were acclimated upon arrival for a week before being assigned randomly to their respective groups based on body weight. Rats (3/cage) were housed in a polypropylene cage and individually identified by numbers on their tails. Each cage was covered with wire bar lid and filtered top (Allentown, NJ, USA). Each individual cage was identified with a cage card indicating project number, test article, dose level, group, and animal numbers. The Harlan T7087 soft cob beddings were used and changed at least twice a week. Animals were provided with fresh water and rodent chow diet # T2018 (Harlan Teklad, 370W, Kent, WA, USA) ad libitum and were housed in a temperature-controlled room (22.2°C) on a 12 h light-dark cycle. All animal experiments were conducted according to the institutional guidelines congruent with guide for the care and use of laboratory animals.

### 2.4. Monoiodoacetate- (MIA-) Induced Experimental Osteoarthritis Model Induction and Treatment

Treatment started a week before MIA injection. Animals (body weight 215–229 g) were randomized into six groups of 10 rats per group as G1 = normal control (no MIA, treated with 0.5% CMC-Na solution), G2 = MIA (MIA injected, treated with 0.5% CMC-Na solution), G3 = MIA + diclofenac (10 mg/kg, Lot # W08B043, Ward Hill, MA), G4 = MIA + UP1306 (400 mg/kg) (Lot # AM14002), G5 = MIA + UP446 (250 mg/kg), and G6 = MIA + composition - UP1306 (400 mg/kg) + UP446 (250 mg/kg) were orally administered with respective treatment. The standardized blends for each treatment groups are defined as UP446 = 1A : 4S, UP1306 = 1A : 2M, and the composition at the given dosage = 0.282A : 0.308S : 0.410M, where “A,” “S,” and “M” stand for* Acacia*,* Scutellaria*, and* Morus*, respectively. On the induction day, isoflurane (Lot #B66H15A, Piramal Enterprise Ltd., Andhra Pradesh, India) anesthetized rats were injected with 0.8 mg of MIA (Lot # A0352046, Acros Organics, New Jersey, USA) in 50 *μ*L saline solution into the intra-articular pocket of left femorotibial (knee) joint using 26 G needle an hour after treatment. Normal control rats were injected with an equal volume of saline. Paw withdrawal thresholds as a result of constant pressure applied to the affected joint as a measure of pain sensitivity were taken once a week using Randall-Selitto Anesthesiometer (IITC, USA) and treatment lasted for 6 weeks. Body weights were measured once a week to calculate the respective weekly dosage of each group. The merit of combining UP1306 and UP446 was then evaluated using Colby's equation [8]. Percent inhibition values were used to determine the calculated efficacy values and compared to the observed percent inhibition values of the composition.

### 2.5. Histopathology Procedures and Evaluations

At necropsy, animals were asphyxiated with CO_2_ and the femorotibial joint was carefully dissected out, fixed in 10% buffered formalin, and sent to Nationwide Histology (Veradale, WA, USA) for further histopathology analysis. The fixed specimens were then decalcified with Calci-Clear Rapid for 1 and a half days and embedded in paraffin. Standardized 5 *μ*m serial sections were obtained at the medial and lateral midcondylar level in the sagittal plane and were stained with hematoxylin and eosin (HE) and Safranin O-fast green to enable evaluation of proteoglycan content. A modified Mankin system [9] was used to score structural and cellular alterations of articular components as indications of disease progression and/or treatment efficacy. The histological analysis was conducted by a certified pathologist who was blinded to the coded specimens at Nationwide Histology.

### 2.6. CTX-II ELISA Assay

CTX-II enzyme-linked immunosorbent assay (ELISA) was performed according to the kit manufacturer's specifications. Briefly, a standard curve was prepared by generating serial dilutions (1 : 2) of CTX-II standard (5000 pg/mL) in Sample Diluent. Rat urine samples were thawed on ice and diluted 1 : 3 with Sample Diluent. 100 *μ*L of samples and standards were added to a 96-well plate precoated with CTX-II antibody. The plate was covered and incubated at 37°C for 2 hours. The liquid was aspirated from each well; then 100 *μ*L of Detection Reagent A working solution was added to each well. The plate was covered and incubated at 37°C for 1 hour. The solution was aspirated from each well and the plate was washed three times with 1x Wash Buffer. 100 *μ*L of Detection Reagent B working solution was added to each well; then the plate was covered and incubated at 37°C for 1 hour. The solution was aspirated from each well and the plate was washed five times with 1x Wash Buffer. 90 *μ*L of Substrate Solution was added to each well; then the plate was covered and incubated at 37°C for 30 minutes. 50 *μ*L of Stop Solution was added to each well, and the optical density at 450 nm was detected using a Tecan Genios plate reader. CTX-II concentration for each sample was determined by multiplying the dilution factor and fitting the value to the standard curve. To offset the variations in urine flow between rats, values were normalized to total urine protein.

### 2.7. Statistical Analysis

Data were analyzed using SigmaPlot (Version 11.0, Systat Software, Inc., San Jose, CA, USA). The results are represented as mean ± standard deviation. Statistical significance among groups was calculated by means of single factor analysis of variance (ANOVA) and by *t*-test. *P* ≤ 0.05 were considered statistically significant. When normality test failed, for nonparametric analysis, data were subjected to Mann–Whitney sum and Kruskal–Wallis one-way ANOVA on ranks for *t*-test and ANOVA.

## 3. Results

### 3.1. Antipain Sensitivity Activity of Compositions in MIA-Induced OA Model

**Figure 1:**
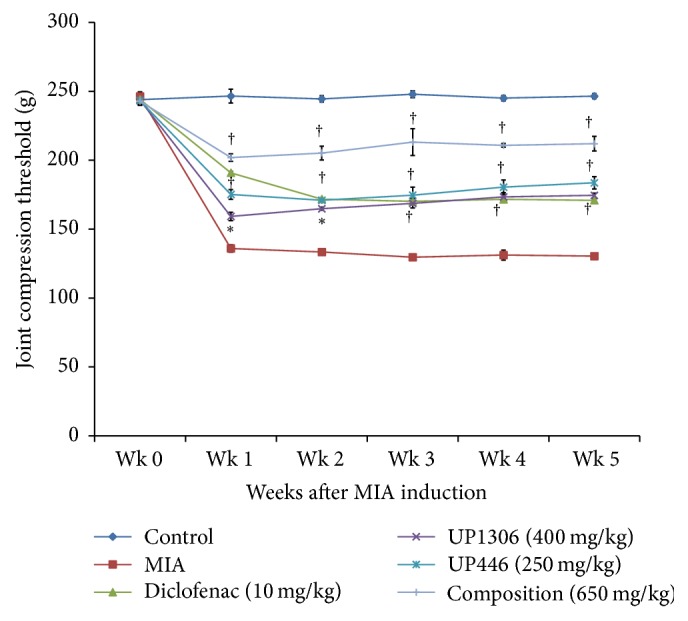
Compression threshold for MIA-injected rats treated with UP1306, UP446, and their composition. OA disease model was induced by intraarticular injection of 0.8 mg/joint MIA to the left femorotibial joint of SD rats (*N* = 10). Rats were treated with diclofenac, UP1306, UP446, and the composition at oral doses of 10 mg/kg, 400 mg/kg, 250 mg/kg, and 650 mg/kg, respectively, for 6 weeks. Compression threshold was assessed every week using Randall-Selitto Anesthesiometer. Data are expressed as mean ± SD. ^*∗*^*P* ≤ 0.00001; ^†^*P* ≤ 0.000001.

Pain sensitivity and inhibition data have been depicted in Figure 1 and Table 1. Rats started showing repose to pain one week after model induction. Vehicle treated MIA rats showed progressive increase in pain sensitivity as exhibited by the mean pain sensitivity values. Compared to the vehicle treated normal controls, these rats showed 44.9, 45.4, 47.7, 46.5, and 47.1% increase in pain sensitivity from week 1 to week 5, respectively. In contrast, all treatment groups showed statistically significant inhibition in pain sensitivity for all the weeks. The highest inhibition in pain sensitivity was observed for rats treated with the composition. These reductions were compared against the vehicle treated group and found as 59.6%, 64.6%, 70.7%, 69.9%, and 70.3% from week 1 to week 5, respectively. Rats given UP446 at 250 mg/kg experienced greater inhibition in pain sensitivity than UP1306 at 400 mg/kg. The observed pain reliefs were statistically significant at each data point examined for both the studies for all the treatment groups. Interestingly, the pain reductions observed for the composition were statistically significant when compared to the diclofenac at each monitored time point (*P* ≤ 0.0001).

### 3.2. Unexpected Pain Reduction Synergistic Activity of a Composition Comprised of* Morus*,* Acacia*, and* Scutellaria* Extracts

The merit of combining UP1306 and UP446 at the indicated dosages for pain relief was also assessed using Colby's equation. In this method, a formulation of two or more materials together will presume to have unexpected synergy when the observed value of a certain end point measurement is greater than or equal to the hypothetically calculated values. In this study, pain sensitivity was used as the end point measurement for synergy determination. As seen in Table 2, the observed pain inhibitions were greater than expected for the composition at each week administered at oral doses of 650 mg/kg.

### 3.3. Improved Histological Findings as a Result of a Composition Comprised of* Morus*,* Acacia*, and* Scutellaria* Extracts in MIA-Induced OA Model

**Figure 2:**
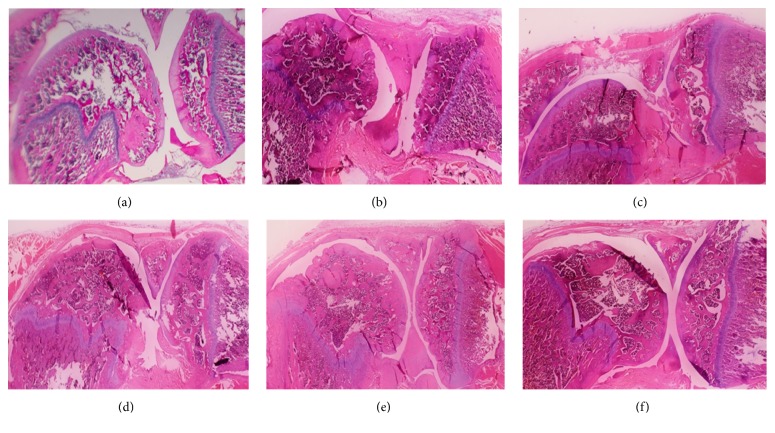
Histopathology images of MIA-induced rats treated with UP1306, UP446, and their composition: the femorotibial joint was carefully dissected out, fixed in 10% buffered formalin, then decalcified with Calci-Clear Rapid for 1 and a half days, and embedded in paraffin. Standardized 5 *μ*m serial sections were obtained at the medial and lateral midcondylar level in the sagittal plane and were stained with hematoxylin and eosin (HE) for each rat. Each treatment group has 10 rats per group. Representative image from one rat of each group has been reported. (a) Normal control; (b) MIA control; (c) MIA + diclofenac (10 mg/kg); (d) MIA + UP1306 (400 mg/kg); (e) MIA + UP446; (f) MIA + composition.

Complementing the pain sensitivity reduction data, statistically significant improvements in articular cartilage matrix integrity were shown as reflected by the modified total Mankin score for animals treated with the compositions and their active constituents. Structural abnormalities and fibrovascular proliferation were also significantly reduced in this group. At necropsy, when the overall structural abnormality (such as cartilage thickening or thinning, surface irregularity, fissure loss, degeneration, ulcerative necrosis, severe disorganization, and chaotic appearance) was assessed, reductions of 38.3, 60.5, and 56.5% were observed for rats treated with UP1306 (400 mg/kg), UP446 (250 mg/kg), and composition (650 mg/kg), respectively, when compared to the vehicle treated MIA rats (Table 2 and Figure 2). Diclofenac showed 51.4% and 57.7% reductions in structural abnormalities and inflammation, when compared to the vehicle treated MIA rats, respectively (Table 3 and Figure 2). The highest inhibition (80.8%) in inflammation and infiltration of inflammatory cells was observed for rats treated with the composition at 650 mg/kg as compared to the 37.7% and 53.8% inhibitions from the constituents UP1306 and UP446, respectively, when compared to the vehicle treated MIA rats (Table 3 and Figure 2). In comparison to the diclofenac treated rats, the rats treated with the composition experienced statistically significant reductions in inflammation (*P* = 0.01). Nonstatistically significant improvements in other structural and cellular changes were also observed for the composition compared to the diclofenac group except in the fibrovascular proliferation where the diclofenac group showed better outcome.

The extent of osteoclast activities and subchondral bone damage were minimal for all the treatment groups. In contrast, various degrees of histopathological changes including cellular degeneration and disorganization of the articular cartilage chondrocytes, depletion and collapse of the intracellular matrix, articular surface irregularities, osteophyte remodeling, and fibrillation of the subchondral bone were observed for vehicle treated MIA rats. These changes are similar to the most common findings in human OA biology [10]. In Safranin O staining, articular cartilage of treatment groups revealed minimum loss of staining intensity indicating its ability to spare cartilage degradation (Table 3 and Figure 2). For instance, reductions of 46.0, 31.0, 59.7, and 57.1% were observed in matrix GAG loss for diclofenac (10 mg/kg), UP1306 (400 mg/kg), UP446 (250 mg/kg), and the composition (650 mg/kg), respectively. Rats in the normal control groups treated with vehicle showed negligible changes in all the examined parameters (Table 3 and Figure 2). Normal structure of the articular cartilage, subchondral bone of both tibia plateaus and femoral bone, and the surrounding joint structure appeared intact in this group of rats.

### 3.4. Significant Reductions in Urine CTX-II Level as a Result of the Composition

Urine CTX-II (C-telopeptide of type II collagen) is a type II collagen biomarker frequently referenced for its close correlation with progression of articular cartilage degradations. After 5 weeks of daily oral treatment, 122.6 and 54.1% reductions in urine CTX-II were observed for rats treated with diclofenac (10 mg/kg) and UP1306 + UP446 (650 mg/kg) (Table 4). While the reduction seemed to appear higher for the diclofenac group (in comparison to the composition), it was not statistically significant due to variations between individual rats. This inhibition of cartilage degradations and hence low level of urine CTX-II were statistically significant for rats treated with the composition. On the other hand, UP1306 and UP446 treated rats showed 10.4 and 7.8% reductions in uCTX-II, respectively.

## 4. Discussion

The present study depicts data from MIA-induced rat OA disease model treated with a composition composed of UP1306 and UP446 for 6 weeks. Significant improvements in articular cartilage protection and pain tolerance were documented. Model induction was confirmed by the significantly increased hypersensitivity and later by histopathological findings and CTX-II data. On the other hand, various degrees of inhibitions in pain sensitivity were observed for rats treated with the composition and its individual components. Statistically significant, up to 70.7%, inhibition in pain sensitivity was observed for the rats treated with the combination of UP1306 and UP446 when compared to the vehicle treated MIA rats. These degrees of pain reductions observed for the combination of the two products were significantly higher at each time point evaluated compared to either of the ingredients. In agreement with the in-life pain relief observations, the cartilage sparing activity was significantly higher for the rats in the combination group compared to either of the constituents. These findings were also matched with the urinary CTX-II level where more than 54% reduction in UCTX-II level was observed for the combination therapy. The merit of formulating these known anti-inflammatory and analgesic products was also confirmed.

OA is an elusive disease with unspecified initial etiology involving articular cartilage, subchondral bone, and synovial membrane. Progressive articular cartilage degradation is the hallmark of OA. Cartilage is the main component of articular structure and consists of chondrocytes that are embedded in a dense and highly organized extracellular matrix (ECM). ECM is synthesized by the chondrocytes and is composed of a collagenous network that primarily contains type II collagen, along with glycosaminoglycans (GAGs) and associated proteoglycans. Articular cartilage degradation occurs as a result of an imbalance in the homeostasis of these fundamental matrix components [11]. This pathogenesis is triggered in part by the action of inflammatory cytokines, primarily interleukin-1 (IL-1) [12, 13], that also mediate the production of proinflammatory mediators (including nitric oxide, NO, and prostaglandin E2-PGE2) and matrix degrading enzymes, aggrecanase and matrix metalloproteinase (MMP). While the catabolic enzymes, MMPs, disrupt collagen fibers [14], members of a disintegrin and metalloprotease with thrombospondin (ADAMTS) family degrade aggrecan and both cases result in the release of GAGs [15]. Therefore, plant extracts with proven anti-inflammatory and/or antiprotease/aggrecanase activity could potentially be administered for their potential cartilage protection activity in OA patients.

The MIA-induced OA disease model in rats is a standardized model most frequently used to mimic the human OA [16]. The model involves inoculation of MIA into a femorotibial joint pocket that induces pain responses in the ipsilateral limb accompanied by progressive cartilage degradation. Intra-articular injection of MIA disrupts chondrocyte glycolysis by inhibiting glyceraldehyde-3-phosphatase dehydrogenase and results in chondrocyte death, neovascularization, and subchondral bone necrosis and collapse, as well as inflammation [17]. These characteristics make the model very attractive for evaluating compounds for their anti-inflammatory, analgesic, and/or potential disease modifying activities as it shares similar disease pathology to the human OA. Previously, both UP1306 and UP446 were tested in multiple animal models mainly focused on their symptomatic relief in inflammation and pain. Both showed good anti-inflammatory and antipain activity (Burnett et al., 2007; Tseng-Crank et al., 2010; Altavilla et al., 2009; [5]). Inflammation plays a central role in osteoarthritis (OA) pathology. Proinflammatory cytokines are known to cause increase of production of protease and aggrecanase which in turn lead to degradation of cartilage. Cartilage degradation is the hallmark of OA. This vicious cycle of OA pathophysiology can be addressed in the MIA model. As a result, we selected this mature model to investigate the effect of UP1306 and UP446 alone or in combination in maintaining articular structural integrity and mitigating pain sensitivity administered orally for 6 weeks.

Coupled with symptoms and biomarkers, histopathological analyses of articular cartilage, synovial membrane, and subchondral bone have been used to evaluate OA disease progression or to measure outcome of therapeutic interventions (Goldring et al., 2000). In the current study, significant improvements in maintenance of the articular structural integrity of rats treated with the composition were observed. These effects were demonstrated in the histopathology data as exhibited by limited loss, degeneration, or necrosis of chondrocytes, smoother articular cartilage surface, deeper and uniform stain of intracellular matrix, and close to normal contour of the subchondral bone. For obvious reasons, this minimal cartilage degradation was also supported by the significant reductions in pain sensitivity where the composition achieved unexpected synergistic impact in reducing OA associated pain. The changes in magnitude of inhibition were computed and it was found that the composition performed 180.1, 128.0, 114.0, 89.1, and 84.9% better than UP1306 and 65.7, 91.5, 85.6, 61.4, and 53.2% more than UP446 at week 1, week 2, week 3, week 4, and week 5, respectively. While the marked inhibition in cartilage degradation and pain sensitivity observed for the combination therapy is unexpected, previously reported individual antipain and anti-inflammatory activity of UP446 and UP1306 could be relevant for this significant outcome (Yimam et al., 2017; Yimam et al., 2012; Yimam et al., 2013; [5]). As recently reported, when rats were treated with oral doses of UP1306 at 300 mg/kg, up to 53.3% reductions in paw edema and up to 54.8% reductions in pain sensitivity in the carrageenan induced rat paw edema model, 34.4% reduction in visceral pain sensitivity in the abdominal constriction test in mice [5], and up to 41% inhibition in pain sensitivity in MIA-induced rat OA model (Yimam et al., 2017) were observed. Statistically significant improvements in articular cartilage matrix integrity and minimal subchondral bone damage were also documented in the MIA model (Yimam et al., 2017). Supplementing these findings, 40% reductions in pain sensitivity and 66% reductions in paw edema in carrageenan induced rat paw edema model, administered orally at 150 mg/kg, 58% reductions in visceral pain sensitivity in abdominal constriction test in mice, administered orally at 100 mg/kg (Yimam et al., 2012), and statistically significant inhibition in hypersensitivity, paw edema, ankle diameter, and paw thickness in the complete adjuvant induced rheumatoid arthritis model in rats, administered at 50 mg/kg, were observed for UP446 (Yimam et al., 2013). Therefore, putting UP446 and UP1306 at specific ratio might have provided a boost for the cartilage protection and antipain activity of their combination.

Furthermore, as demonstrated by the urine CTX-II, statistically significant reduction in the level of uCTX-II was also observed for rats treated with the composition. Here, neither UP1306 nor UP446 treated rats reached a significant level of reductions when administered alone. In comparison, the composition resulted in 48.8% and 50.2% greater reductions in uCTX-II than its constituents UP1306 and UP446, respectively. These findings confirm that combining these plant extracts together could result in a profound boost to their cartilage sparing activity and hence alleviation of pain. Substantiating this statement in human clinical studies, urine CTX-II levels were well aligned with cartilage degradation and associated pain in OA patients [4, 18]. For example, urinary CTX-II concentrations were found elevated and associated with knee pain and function in subjects undergoing anterior cruciate ligament reconstruction [18]. In these patients, decreased uCTX-II concentrations were correlated with decreased knee pain and improving function providing meaningful prognosis [18]. Similarly, in a cross-sectional evaluation of biochemical markers of bone, cartilage, and synovial tissue metabolism in patients with knee osteoarthritis, uCTX-II were found to be significantly increased with corresponding disease severity and were correlated with changes in joint space narrowing [4].

Separately, prenylated flavonoids from* M. alba*, free-B-ring flavonoids from* S. baicalensis*, and flavans from* A. catechu* have traditionally been used for various human ailments. Their long history of safe human consumption makes these botanicals best fitting for OA management that requires chronic daily intake by patients. Individual plants have showed some form of protease and aggrecanase inhibition activities. For instance, prenylated flavonoids extracted from* M. alba* have shown inhibition of the catabolic enzymes ADAMTS1 [19] and matrix metalloproteinase (MMP-1 and MMP-3) (Kim et al., 2011; [20]). Catechin, the major flavan of* Acacia*, also inhibited the degradation of human and bovine cartilage proteoglycan and type II collagen [21] and hindered IL-*β* induced cartilage proteoglycan degradation and expression of matrix metalloproteinases (MMP1 and MP-13) in human chondrocytes [22]. Similarly, baicalin, the primary active component of* Scutellaria*, demonstrated inhibition of MMP-3 and MMP13 expressions [23] as well as ADAMTS-4, ADAMTS-5, cathepsin K, and cathepsin B [24] in IL-1 and TNF-*α* stimulated human chondrocyte. However, the use of these extracts in combination for cartilage protection or associated symptoms has never been reported. As a matter of fact, there has not been any report in the literature for any of the disclosed extracts to reduce urinary CTX-II. Hence, findings in the current report could not be directly compared with previous formulations of the same kind.

Considering the multifactorial nature of OA, it has previously been suggested that the ability to slow the progression of articular cartilage degeneration is greater with a combination therapy than that of any single component alone [25]. The composition of bioflavonoid standardized extracts from* Scutellaria baicalensis*,* Acacia catechu*, and* Morus alba* may suit very well in this category. In fact, when the merit of formulating these three plant materials was tested, an unexpected synergy in alleviating pain sensitivity was observed from the combination of these three plant materials that exceeded the predicted result based on simply summing the effects observed for each of its constituents. Furthermore, significance of urinary CTX-II level reductions was achieved only for the standardized blend consisting of these three extracts. Overall, (A) reduction in urine CTX-II level, (B) articular cartilage protection, and (C) unexpected significant reductions in pain sensitivity were the fundamental findings of this study. Clinical and preclinical literature searches failed to produce previous reports of either of the constituents to reduce urine CTX-II levels in OA. This signifies the novelty of the composition in maintaining articular structural integrity as reflected by the reduced uCTX-II level accompanied by minimal pain sensitivity. We believe that these medicinal plants may have complementary effects on each other in preventing articular cartilage degradation and mitigating associated pain which could be translated to improved joint mobility and function.

The current study was not without a limitation. While improved articular cartilage protection and pain tolerance are considered as the significant findings of the study, the mechanisms by which the composition resulted in these outcomes were not investigated in the present study. The reduced urinary CTX-II level is simply an indication for the cartilage sparing activity of the composition. Hence, there still is a need for additional studies to determine the specific pathways that could be affected as a result of treatment with the composition. Impact of treatment in association with proinflammatory cytokines and cartilage degrading enzymes (such as proteases and aggrecanases) will also require further confirmation.

Clinically, both UP446 and UP1306 have been tested separately in randomized placebo and active controlled human clinical trials administered at daily oral doses of 250–500 mg/day and 400 mg/day, respectively, for a duration of 90 days. While UP446 resulted in statistically significant improvement in range of motions, reduction in pain score and stiffness compared to the active comparator [6] and marked changes were observed for those parameters for UP1306 compared to baseline (manuscript under review in the Nutrition Journal). Most importantly, in the UP1306 clinical trial, statistically significant 8.9% reduction in uCTX-II was observed for UP1306 treated subjects compared to the placebo group. A quick onset, as early as 3 days of improvement in stiffness and 5 days in pain relief was also reported in another clinical trial for UP446 [7]. Hence, the formulation of these products may provide significant clinical implications in both symptomatic relief and cartilage protection activity. Given the long history of safe usage of individual plants and significant safety and efficacy data of UP446 and UP1306, it can easily be predicted that the future success of their combination lies in the clinical efficacy.

## 5. Conclusion

We have evaluated the efficacy of a composition comprised of* Acacia* (A),* Scutellaria* (S), and* Morus* (M) extracts at the ratio of 0.282A : 0.308S : 0.410M administered orally to MIA-induced osteoarthritis disease model in the rats. Significant reduction in urinary CTX-II, synergistic pain alleviation, and maintenance of articular structural integrity were observed as a result of oral treatment of their composition. No sign suggestive of toxicity was observed during study. These findings suggest that the standardized blend of these extracts could potentially be considered as an alternative therapy from natural sources for the treatment of OA and its associated symptoms.

## Figures and Tables

**Table 1:** Percent changes in pain sensitivity for MIA-induced rats treated with UP1306, UP446, and their composition.

Group	Dose (mg/kg)	*N*	Wk 1	Wk 2	Wk 3	Wk 4	Wk 5
*% increase*							
MIA	10	10	44.9^†^	45.4^†^	47.7^†^	46.5^†^	47.1^†^

*% inhibition*							
Diclofenac	10	10	49.6^†^	34.5^†^	34.3^†^	35.5^†^	34.9^†^
UP1306	400	10	21.1^*∗*^	28.3^*∗*^	33.0^†^	37.0^†^	38.0^†^
UP446	250	10	35.5^†^	33.8^†^	38.1^†^	43.3^†^	45.9^†^
Composition^‡^	650	10	59.6^†^	64.6^†^	70.7^†^	69.9^†^	70.3^†^

^*∗*^ *P* ≤ 0.00001 verses MIA; ^†^*P* ≤ 0.000001 verses MIA or normal control; % increase = ((mean normal control − mean MIA)/mean normal control) *∗* 100; % inhibition = ((mean treatment − mean MIA)/(mean normal control − mean MIA)) *∗* 100. ^‡^Composition: UP1306 + UP446.

**Table 2:** Unexpected synergistic effect of UP1306 and UP446 combination in reducing pain sensitivity.

Pain inhibition synergy calculation (%)						
Weeks after MIA		Wk 1	Wk 2	Wk 3	Wk 4	Wk 5
UP446 (*x*)	250 mg/kg	28.9	28.1	34.7	37.6	40.8
UP1306 (*y*)	400 mg/kg	17.1	23.6	30.1	32.1	33.8

Equation	(*x* + *y*)	46.0	51.7	64.8	69.7	74.6
	(*xy*)/100	4.9	6.6	10.4	12.1	13.8

Expected	[(*x* + *y*)−(*xy*)/100]	41.1	45.1	54.4	57.6	60.8

Observed	Composition^†^ (650 mg/kg)	47.9	53.8	64.4	60.7	62.5

(i) ^†^Composition: UP1306 + UP446; (ii) UP446 (*x*); UP1306 (*y*); Colby's equation: [(*x* + *y*)−(*xy*)/100]. Data interpretation: a formulation of two or more materials together will presume to have unexpected synergy when the observed value of a certain end point measurement is greater than or equal to the hypothetically calculated values. That is, observed ≥ expected = synergy. Observed = composition pain inhibition; expected = theoretical calculated pain inhibition value.

**Table 3:** Modified Mankin scoring system for histopathological findings for MIA-induced rats treated with UP1306, UP446, and their composition.

Group	Dose (mg/kg)	MIA mg/joint	*N*	Structural abnormality	Bone at articular surfaces	Inflammation/cellular infiltration	Fibrovascular proliferation	Matrix GAGs
Control−	0	0	10	1.15 ± 0.61^†^	0.90 ± 0.58^†^	0.75 ± 0.34^*∗*^	1.35 ± 0.63^*∗*^	1.36 ± 0.67^†^
MIA+	0	0.8	10	2.53 ± 0.79	1.90 ± 0.70	1.30 ± 0.68	2.40 ± 1.02	2.68 ± 0.76
Diclofenac	10	0.8	10	1.23 ± 0.36^‡^	0.70 ± 0.24^‡^	0.55 ± 0.15^†^	1.55 ± 0.69^*∗*^	1.44 ± 0.38^‡^
UP1306	400	0.8	10	1.56 ± 0.38^†^	1.35 ± 0.50	0.81 ± 0.50	1.60 ± 0.83	1.85 ± 0.37^*∗*^
UP446	250	0.8	10	1.00 ± 0.40^‡^	1.05 ± 0.47^*∗*^	0.60 ± 0.20^*∗*^	1.75 ± 0.64	1.08 ± 0.34^‡^
Composition^*₰*^	650	0.8	10	1.10 ± 0.51^‡^	0.70 ± 0.60^†^	0.25 ± 0.25^‡^	1.85 ± 0.71	1.15 ± 0.46^‡^

^*∗*^ *P* ≤ 0.05; ^†^*P* ≤ 0.001; ^‡^*P* ≤ 0.0001. ^*₰*^Composition: UP1306 + UP446. MIA: monoiodoacetate. Structural abnormality (0–6): cartilage thickness/thinning, frayed irregular surface/fissure loss, degeneration, ulcerative necrosis/fragmentation, and severe disorganization/chaotic appearance; bone at the articular surfaces (0–6): subchondral bone thickness/volume and density, osteoclastic activity, and subchondral bone damage; inflammation/cellular infiltration (0–6): cellular infiltration/inflammation and proliferation, hypercellular and cluster/hypocellular; fibrovascular proliferation (0–6): fibrovascular proliferation replacing periarticular/capsular/bone parts (pannus), condyle and/or tibial plateau, meniscus reduction, fusion, and adhesion; matrix GAGs (0–6): matrix GAGs reduction: radial, interterritorial to pericellular loss of staining, femoral condyle/tibial plateau-integrity, and thickness of articular cartilage.

**Table 4:** uCTX-II normalized to total protein at week 5.

Group	Dose (mg/kg)	*N*	uCTX-II (ng/L) (mean ± SD)	Total protein (g/L) (mean ± SD)	Ratio (CTX-II/protein)	*P* values versus MIA+
Control−	0	5	365 ± 145	3.08 ± 1.02	118.39	0.332
MIA+	0	10	635 ± 233	6.46 ± 2.64	98.30	—
Diclofenac	10	8	304 ± 233	4.72 ± 1.23	64.41	0.017
UP1306	400	10	607 ± 237	7.91 ± 2.57	76.74	0.129
UP446	250	10	614 ± 148	7.56 ± 2.34	81.22	0.103
Composition^†^	650	8	489 ± 172	7.66 ± 2.37	63.84	0.003

(i) ^†^Composition: UP1306 + UP446; (ii) % inhibition = ((mean treatment − mean MIA)/(mean normal control − mean MIA)) *∗* 100.
